# Cardiac fibrosis and dysfunction in experimental diabetic cardiomyopathy are ameliorated by alpha-lipoic acid

**DOI:** 10.1186/1475-2840-11-73

**Published:** 2012-06-19

**Authors:** Chun-jun Li, Lin Lv, Hui Li, De-min Yu

**Affiliations:** 1 Key Laboratory of Hormone and Development (Ministry of Health), Metabolic Disease Hospital & Tianjin Institute of Endocrinology, Tianjin Medical University, 66# TongAn Road, Heping District, 300070, Tianjin, China; 2 Translational Research Center, Institute for Translational Medicine and Therapeutics, Perelman School of Medicine at University of Pennsylvania, Philadelphia, PA, USA

**Keywords:** Alpha-Lipoic acid, Cardiac fibrosis, Extracellular matrix remodeling, Mitochondrial oxidative stress

## Abstract

**Background:**

Alpha-lipoic acid (ALA), a naturally occurring compound, exerts powerful protective effects in various cardiovascular disease models. However, its role in protecting against diabetic cardiomyopathy (DCM) has not been elucidated. In this study, we have investigated the effects of ALA on cardiac dysfunction, mitochondrial oxidative stress (MOS), extracellular matrix (ECM) remodeling and interrelated signaling pathways in a diabetic rat model.

**Methods:**

Diabetes was induced in rats by I.V. injection of streptozotocin (STZ) at 45 mg/kg. The animals were randomly divided into 4 groups: normal groups with or without ALA treatment, and diabetes groups with or without ALA treatment. All studies were carried out 11 weeks after induction of diabetes. Cardiac catheterization was performed to evaluate cardiac function. Mitochondrial oxidative biochemical parameters were measured by spectophotometeric assays. Extracellular matrix content (total collagen, type I and III collagen) was assessed by staining with Sirius Red. Gelatinolytic activity of Pro- and active matrix metalloproteinase-2 (MMP-2) levels were analyzed by a zymogram. Cardiac fibroblasts differentiation to myofibroblasts was evaluated by Western blot measuring smooth muscle actin (α-SMA) and transforming growth factor–β (TGF-β). Key components of underlying signaling pathways including the phosphorylation of c-Jun N-terminal kinase (JNK), p38 MAPK and ERK were also assayed by Western blot.

**Results:**

DCM was successfully induced by the injection of STZ as evidenced by abnormal heart mass and cardiac function, as well as the imbalance of ECM homeostasis. After administration of ALA, left ventricular dysfunction greatly improved; interstitial fibrosis also notably ameliorated indicated by decreased collagen deposition, ECM synthesis as well as enhanced ECM degradation. To further assess the underlying mechanism of improved DCM by ALA, redox status and cardiac remodeling associated signaling pathway components were evaluated. It was shown that redox homeostasis was disturbed and MAPK signaling pathway components activated in STZ-induced DCM animals. While ALA treatment favorably shifted redox homeostasis and suppressed JNK and p38 MAPK activation.

**Conclusions:**

These results, coupled with the excellent safety and tolerability profile of ALA in humans, demonstrate that ALA may have therapeutic potential in the treatment of DCM by attenuating MOS, ECM remodeling and JNK, p38 MAPK activation.

## Background

Diabetes is one of the major health problems around the world. It is estimated that the number of diabetic patients will increase from 135 million in 1995 to 300 million by the year 2025 [1]. Diabetic patients have a 2- to 5-fold increased risk of developing heart failure, one of the most greatest contributors to morbidity and mortality [2]. Diabetic cardiomyopathy (DCM) is defined as the ventricular dysfunction that occurs in diabetic patients independent of another cause, such as coronary artery disease or hypertension [3,4]. The term “DCM” was initially introduced by Rubler in 1972 based upon post-mortem finding in diabetic adults who had HF in the absence of other co-morbid conditions [5]. The term now also embraces diabetic individuals with diastolic dysfunction. Somaratne recently reported that 56 % of diabetic patients had DCM [6]. Although the etiology of DCM is poorly understood, the pathophysiology of DCM is believed to be multifactorial. Existing evidences suggests that persistent hyperglycemia-induced MOS is a significant contributor to DCM [7,8]. Indeed, the activity of several anti-oxidant enzymes is decreased in the diabetic heart in both rats and humans [9].

Cardiac fibrosis is a major feature of DCM [10]. In this condition, an over-production of extra cellular matrix (ECM) protein leads to increased myocardial stiffness and consequent cardiac dysfunction, ultimately resulting in cardiac failure. Therefore, assessment of the balance between ECM synthesis and degradation is a good way to predict the development of diabetes-induced cardiac fibrosis. Elucidating the underlying mechanism and signaling pathways involved in ECM remodeling is also, therefore, of great importance.

The harmful effects of oxidative stress on the diabetic heart are well established. These include abnormal gene expression, altered signal transduction, and activation a number of secondary messenger pathways which lead to cell death and cardiac fibrosis [11-13]. Given the central role of oxidative stress in the pathogenesis of diabetes and diabetic cardiomyopathy, there is growing interest in the use of antioxidants as a complementary therapeutic approach. Numerous studies demonstrated that ameliorating oxidative stress through antioxidant treatment might be an effective strategy for reducing DCM [14,15]. However, traditional antioxidant vitamin E and C clinical trials have failed to demonstrate relevant clinical benefits on cardiovascular disease [7]. Thus there is an urgent need for clinically effective antioxidants. ALA (also known as 1, 2-dithiolane-3-pentanoic acid and thioctic acid) is a naturally occurring compound involved in mitochondrial dehydrogenase reactions and has recently gained a considerable amount of attention as a novel antioxidant. While the beneficial role of ALA in improving diabetic neuropathy has been extensively studied and clinically approved, the effect of ALA on the development and progression of DCM has not yet to be determined. Research studies have demonstrated that intraperitoneal administration of ALA to STZ diabetic Wistar rats normalizes thiobarbituric acid reactive substances (TBARS) level in plasma, retina, liver, and pancreas [16]. Furthermore, in cardiovascular disease, dietary supplementation with ALA has been successfully employed in a variety of in vivo models: ischemia–reperfusion, heart failure, and hypertension [17].

Our previous study [18] and Bojunga [19] both showed that ALA can prevent inappropriate apoptosis in the hearts of diabetic rats. Therefore, in the present study, we extended this work by examining the molecular mechanisms underlying the protective effects of ALA against DCM. This was done by evaluating MOS, cardiac ECM synthesis and degradation and related signaling pathway activities in ALA-treated STZ-induced diabetic rats. To our knowledge, this is the first in vivo study that investigates the effect of ALA treatment on cardiac ECM remodeling in a diabetic rat model. The results show the potential of ALA in the treatment of DCM.

## Materials and methods

### Animals and procedures

The experimental and feeding protocols were approved and in accordance with the laws and regulations controlling experiments on live animals in China and the Asian Convention for the Protection of Vertebrate Animals used in Experimental and Other Scientific Purposes. Eight week old male Wistar rats, weighing 200 to 250 g, were purchased from the Department of Experimental Animals in Peking (China). They were maintained on 12 h light/dark cycle at 21 °C and fed with a standard chow and were given access to tap water *ad libitum*. Rats were rendered diabetes by a single tail intravenous injection of STZ (45 mg/kg, Sigma) dissolved in 0.1 M sodium citrate buffer, pH 4.5 at 4 °C. The STZ induced rat model is a well established model for the study diabetic cardiomyopathy [20]. Insulin supplementation in STZ-induced diabetic animals leads to an improvement of left ventricle (LV) function, indicating the role of hyperglycemia in cardiac dysfunction in this model [21]. Only rats with blood glucose levels ≥16.7 mmol/l 5 days after STZ injection were used in this study. Age-matched non-diabetic Wistar rats were injected with sodium citrate buffer and used as controls. After 1 week following injection STZ, rats were treated with either ALA (100 mg/kg per day i.p.) or vehicle for 11 weeks. The animals were randomly divided into 4 groups, each with 8 rats: normal control (NC), ALA treated control (NC + ALA), non-treated diabetes (STZ) and ALA treated diabetes (STZ + ALA). Rats were sacrificed at 12 weeks after STZ injection.

### Cardiac function evaluation

At the end of the experiment, rats were anesthetized with chloral hydrate (100 mg/kg, i.p.), and cardiac function was determined by invasive hemodynamic evaluation methods. A pressure tip-catheter was positioned into the left ventricle (LV) to record LV pressure-data changes representative of cardiac systolic and diastolic performance. LV diastolic function was quantified by the left ventricular end-diastolic pressure (LVEDP) and maximum rate of fall of left ventricle pressure (-dP/dtmax). LV systolic function was quantified by the left ventricular end-systolic pressure (LVSP) and maximum rate of rise of left ventricle pressure (+dP/dtmax). (BL-420E + system, Tai-meng Instrument Inc., Sichuan, China).

### Tissue preparation

The heart was excised from the chest, trimmed of atria and large vessels and weighed. First, a mid left ventricular (LV) section was cut perpendicularly along the longitudinal axis and fixed in phosphate-buffered 20 % formaldehyde. Histological paraffin-embedded sections (5 μm) were then prepared for Sirius red staining. Some hearts tissue was used for the immediate extraction of mitochondria and some was rapidly frozen in liquid nitrogen and stored at -80 °C for subsequent assay.

### Sirius red staining for interstitial collagen content measurements

LV sections were stained with Sirius Red (Polyscience, Inc, Warrington, PA) to measure interstitial fibrosis. Interstitial collagen was quantified at a final magnification of 200× with a polarized microscope (Olypus, America) connected to a video camera (Nikon, Japan). Perivascular fibrosis was not assessed. The content of interstitial collagen (expressed as the fractional area of the entire cross-section) was averaged over nine fields selected across the septal wall and free wall.

### Measurements of mitochondrial oxidative biochemical parameters

Briefly, hearts were excised and washed in cold PBS and then homogenized in cold lysis buffer supplemented (250 mM/L sucrose, 3 mM/L HEPES and 0.5 mmol/L EDTA pH 7.4). The homogenate was centrifuged twice at 750 g at 4 °C for 10 min and debris discarded. The supernatant from the second spin was removed to fresh tubes and centrifuged twice at 10,000 g at 4 °C for 10 min. The final mitochondrial pellet was resuspended in lysis buffer. The content of malondialdehyde (MDA) in the myocardium mitochondrion was assayed using thiobarbituric acid, and the content of reduced/oxidized glutathione ratio (GSH/GSSG), and the activities of superoxide dismutase (SOD) in the myocardium mitochondria was measured by spectophotometric assays. All procedures were followed by the manufacturer’s instructions. (Jiancheng Inc., Nanjing, China).

### Western blot analysis

The left ventricular tissue of rat hearts were extracted as above and protein concentration was measured using the Bio-Rad protein assay (BioRad, Richmond, CA). Approximately 50 μg protein was loaded onto 8 % or 10 % SDS-PAGE and transferred to a nitrocellulose membrane. The membranes were blocked in 5 % dried milk in Tris-buffered saline (TBS) containing 0.1 % Tween 20 for 1 h, and subsequently incubated overnight with primary antibodies diluted in 5 % dried milk–TBS containing 0.1 % Tween 20. The primary antibodies were: JNK, p38 and ERK (Cell Signaling); TGF β1 and β-Actin (Santa Cruz biotechnology); TIMP-2 (Calbiochem, Bad Soden, Germany); α-SMA, type I and II collagen (abcam USA). Membranes were washed in TBS containing 0.1 % Tween 20 and incubated for 1 h with secondary antibodies (Santa Cruz biotechnology). Bands were visualized using an enhanced chemiluminescense detection kit (Pierce Co.). Quantification of bands was done by gel densitometry with gel image analysis system (American UVP Co).

### Gelatin Zymogram

Heart tissue extracts were loaded onto 8 % SDS-PAGE gels containing 1 mg/ml gelatin under non reducing conditions and were run at 120 V for 90 min together with molecular weight standards. Gels were then washed four times in zymogram renaturing buffer (2.5 % Triton X-100) and incubated overnight in zymogram development buffer (50 mM Tris-HCl, PH 7.5, 200 mM NaCl, 5 mM Cacl2, 0.02 % NaN3). Gels were then stained with Coomassie Blue R-250 followed by destaining with 40 % (v/v) Methanol, 10 % (v/v) Acetic Acid and 50 % water. Clear bands of MMP activity were visible against the blue background.

### Statistical analysis

All statistical operations were performed on a personal computer with the software program SPSS v11.5. Data were collected from repeated experiments and are presented as mean ± SD. One-way ANOVA was used to compare the difference in the 4 groups and Student Newman Keuls Test (SNK) was used for between any 2 groups statistical analysis. P < 0.05 was considered statistically significant.

## Results

### General features

STZ-induced diabetic animals exhibited increased blood glucose levels (28.4 ± 2.8 vs 6.4 ± 0.2 mmol/L) along with decreased body weight (273 ± 45 vs 480 ± 35 g). Diabetic animals also have increased the heart to body weight ratio levels compared to normal control rats (3.25 ± 0.25 vs 2.62 ± 0.16 mg/g). The ALA treatment significantly decreased the heart to body weight ratio, although not back to the normal level in NC group without altering the body weight and blood glucose in diabetic animals; while the ALA treatment in NC animals did not make any change to body weight, heart weight and glucose level, indicating the ALA is well tolerated by the animals and the effects in STZ groups are diabetic specific (Table 1).

**Table 1:** Characteristics of rats at the end of the study (12 weeks)

**Group**	**n**	**Glycemia (mmol/l)**	**Body weight (g)**	**Heart wt/body wt (mg/g)**
NC	8	6.44 ± 0.24	480 ± 35	2.62 ± 0.16
NC + ALA	8	6.26 ± 0.19	492 ± 35	2.60 ± 0.18
STZ	8	28.41 ± 2.84*	273 ± 45*	3.25 ± 0.25*
STZ + ALA	8	26.25 ± 3.49*	277 ± 20*	3.06 ± 0.23*^#^

Eight rats per group. Data are presented as mean values ± standard deviation.

*p < 0.05 vs. NC group; # p < 0.05 vs. STZ group.

### ALA ameliorates diabetes-induced cardiac dysfunction

**Figure 1:**
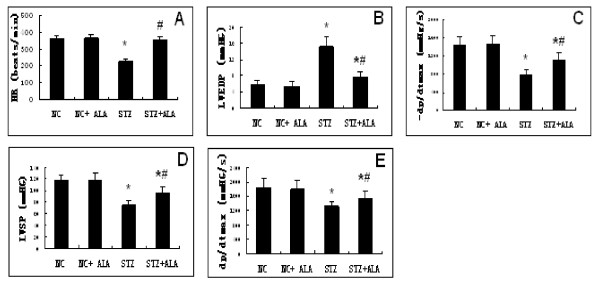
**Diabetes-induced left ventricular dysfunction were improved by ALA treatment.** Cardiac dysfunction were evaluated by measuring (**A**) heart rate (HR), (**B**) left ventricular end-diastolic pressure (LVEDP), (**C**) maximum rate of fall of left ventricle pressure (-dP/dtmax), (**D**) left ventricular end-systolic pressure (LVSP), (**E**) maximum rate of rise of left ventricle pressure (+dP/dtmax). Eleven weeks of STZ-induced diabetes was associated with decrease in HR, LVSP and ± dp/dtmax and an increase in LVEDP, which were significantly improved by ALA treatment for 11 weeks. Results are presented as mean values ± standard deviation. *p < 0.05 vs Control group, #p < 0.05 vs. STZ group, n = 8 per group.

To assess cardiac function, we positioned a pressure detecting catheter into the carotid artery and the LV. After 11 weeks of established diabetes, heart rate (HR) was significantly decreased in STZ group compared to the NC group. This is consistent with diabetes associated dysfunctional cardiac autonomic neuropathy. ALA treatment significantly reversed the HR to the control value. The STZ group also exhibited markedly impaired diastolic function as indicated by an increase in LVEDP and a decrease in -dp/dtmax. There was also impairment of systolic function as shown by a decrease in LVSP and +dp/dtmax. This impaired cardiac function was partially restored by ALA treatment (Figure 1).

### ALA ameliorates diabetes-induced cardiac fibrosis

**Figure 2:**
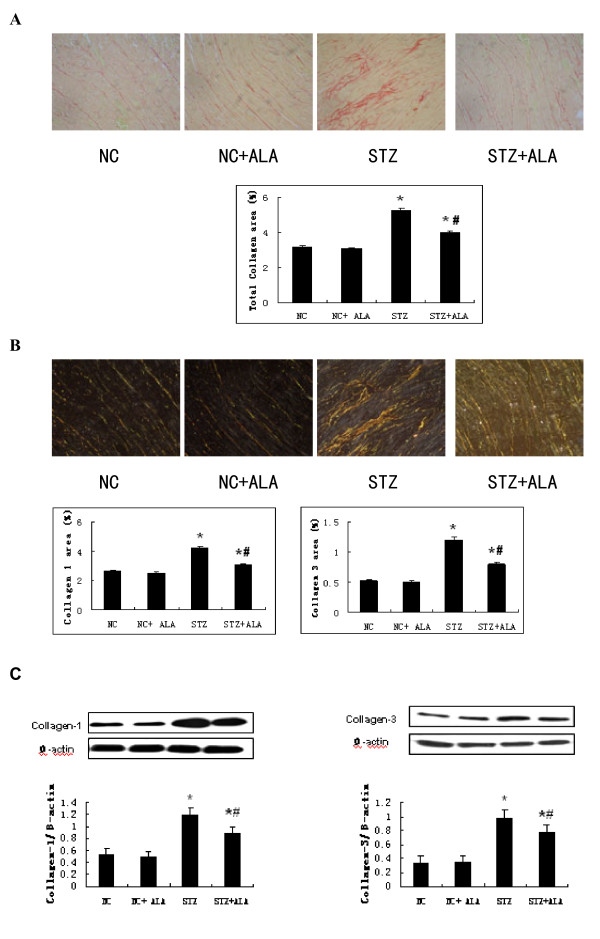
**Diabetes induced cardiac fibrosis.** (**A**) The red color of Sirius red staining under the common microscope indicates total collagen deposition, representative images are from NC, NC + ALA, STZ and STZ + ALA (200 × magnification). (**B**) Type I and II collagen deposition were shown by orange and green color under the polarized light respectively, representative images are from NC, NC + ALA, STZ and STZ + ALA. (**C**) The protein level of type I and II collagen were determined by western blot, β-Actin was used as loading control. Bar graph, density analysis results from 8 rats per group. Results are mean values ± standard deviation. *p < 0.05 vs Control group, #p < 0.05 vs. STZ group.

The total collagen fraction (as determined by red staining) (Figure 2A) was higher in the STZ group compared to NC group, indicating the presence of cardiomyopathy in the diabetic animals. To specifically identify the types of collagen content deposited in the interstitial area, we used polarized light to observe collagen Type I (orange staining) and Type III (green staining). The deposition of both types of collagen was increased in STZ group compared to the NC group (Figure 2B), which is consistent with our Western blot results (Figure 2C). ALA treatment of STZ-induced diabetic rats greatly reduced the deposition of these compounds (Figure 2).

### ALA ameliorates diabetes-induced myocardial MOS

**Figure 3:**
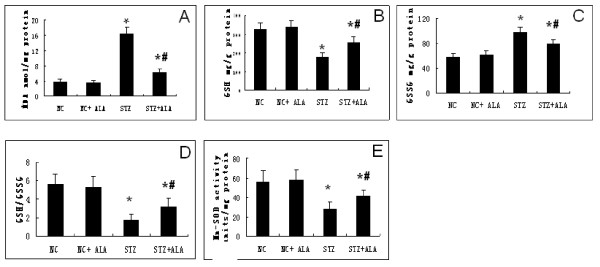
**ALA treatment ameliorated diabetes-induced myocardial mitochondrial oxidative stress.** Myocardial MOS were determined by measuring (**A**) malondialdehyde (MDA) (**B**) endogenous antioxidants reduced glutathione (GSH) (**C**) oxidized glutathione (GSSG) (**D**) the ratio of GSH and GSSG (**E**) superoxide dismutase (SOD) activity. Results are mean values ± standard deviation. *p < 0.05 vs Control group, #p < 0.05 vs. STZ group.

As shown in Figure 3, there were considerably increased MDA levels, a decreased GSH/GSSG ratio and attenuated activity of SOD in myocardial mitochondria of diabetic rats compared to control rats. Interestingly, all these changes were significantly ameliorated by ALA treatment. This indicates that ALA administration can improve diabetes-induced MOS damage.

### ALA decreases the TGF-β, α-SMA, and TIMP-2 expression and increases cardiac active- and pro- MMP-2

**Figure 4:**
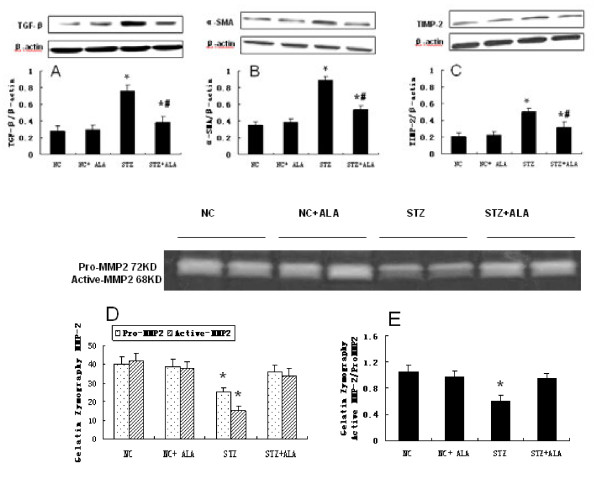
**ALA treatment decreased the TGF-β,α-SMA, and TIMP-2 expression and increased cardiac active- and pro- MMP-2 levels.** The protein expression of TGF-β (**A**), α-SMA (**B**), and TIMP-2 (**C**) were determined by Western blot with specific antibodies as indicated, β-Actin was used as loading control. Cardiac active- and pro- MMP-2 (**D**/**E**) levels were assayed by zymogram. Representative image, bar graph, density analysis results from 8 hearts per group. Data represent mean ± standard deviation. *p < 0.05 vs Control group, #p < 0.05 vs. STZ group.

To evaluate the role of ALA in maintaining extracellular matrix homeostatsis and the preventing cardiac fibrosis, TGF-beta, α-SMA and TIMP-2 were determined as marker of ECM synthesis, while MMP-2 was assayed as an indicator of ECM degradation. STZ-treated diabetic rats had significantly increased LV expression of TGF-βand α-SMA (Figure 4A, 4B), consistent with enhanced ECM synthesis contributing to the increase in cardiac collagen concentration (Figure 2). ALA treatment for 11 weeks significantly decreased LV expression of TGF-β and α-SMA in the diabetic rats, but had no effect on their expression in control rats. As indicator of extracellular matrix degradation, TIMP-2 was shown to be significantly increased in STZ-induced diabetic rats (Figure 4C), consistent with significantly decreased cardiac gelatinolytic activity of active- and pro- MMP-2 levels (Figure 4D) similarly, there was a marked decrease in gelatinolytic activity of active MMP-2 levels (Figure 4E, active MMP-2/Pro-MMP-2). There was no change in the MMP-9 expression and activity in the heart tissues of diabetic rats (data not shown). ALA treatment was able to significantly decrease LV TIMP-2 expression and MMP-2 gelatinolytic activity, but had no markedly effects on basal LV TIMP-2 expression or MMP-2 gelatinolytic activity over the 11 weeks treatment period.

### ALA reduced diabetes-induced MAPK activation

**Figure 5:**
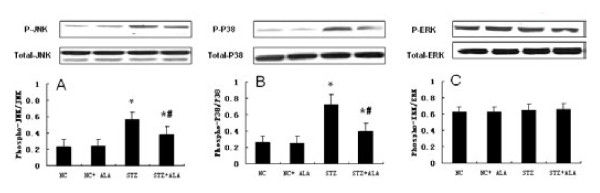
**ALA Reduces diabetes-induced myocardial activation of JNK and P38.** Total protein was obtained from the hearts of STZ-induced diabetic rats and vehicle rats. Phosphorylated and total JNK, P38 and ERK were determined by Western blot with specific antibodies as indicated. The extent of JNK (**A**), P38 (**B**) and ERK (**C**) phosphorylation was quantified by phosphorylated protein/total protein. Representative image, bar graph, density analysis results from 8 hearts per group. Data represent mean ± standard deviation. *p < 0.05 vs Control group, #p < 0.05 vs. STZ group.

To further assess the signaling pathways potentially involved in the cardiac remodeling process induced by diabetes, we examined key components of the MAPK pathways in the treatment groups. As shown in Figure 5, there was marked increase the JNK (Figure 5A) and p38 MAPK (Figure 5B) activation in the heart tissues of diabetic rats compared to the NC group, ALA treatment of diabetic rats for 11 weeks significantly mitigated JNK and p38 MAPK activation. There was no change in the ERK activation in the heart tissues of diabetic rats compared to the NC group; and ALA treatment of diabetic rats has no significant effect on ERK activation (Figure 5C).

## Discussion

Cardiac failure is a leading cause of the mortality in diabetic patients, and is, in part due to DCM. Given the important contributory role of oxidative stress in DCM, antioxidant supplementation has been proposed as an intervention. The present study explored the protective role of ALA on DCM in the STZ-induced diabetic rat model.

Our results demonstrated that STZ injection successfully induced diabetes and DCM as indicated by a decrease in heart rate, increase in cardiac dysfunction and increased collagen deposition in heart tissues. Interestingly, all of these cardiac abnormalities were improved by the administration of ALA. As expected, we have also found the evidence of increased cardiac fibrosis, as suggested by increased TGF-β, α-SMA, TIMP-2 and reduced MMP-2 activity in the diabetic rats, with all these being amelioration by ALA treatment. The observation of attenuated MPAK signaling pathway activation (JNK, p38 MAPK) after ALA administration confirmed our hypothesis that reduced MPAK signaling may contribute to the protective effect of ALA. These findings suggest that ALA treatment may represent a realistic strategy for limiting the progression of cardiac fibrosis associated with diabetes.

ROS production is thought to be an important contributing factor in the development and the progression of diabetic cardiomyopathy [7-9,22]. Inhibition of ROS by over-expression of antioxidant enzymes using transgenic methods in the heart reversed diabetic cardiomyopathy in animal models of both type 1 and type 2 diabetes [3,13,23-25]. However, these methods currently are not practical in clinical practice. In contrast, ALA has been safely used in the clinical management of symptomatic diabetic neuropathy in Germany for more than 30 years. Recently, ALA has been shown to have beneficial effects on treatments of diabetic complications by decreasing ROS generation and increasing glutathione and SOD activity [26]. These results support our experimental data, demonstrating that ALA treatment reduces cardiac dysfunction and remodeling via inhibiting MOS.

Evidence from human and experimental models of diabetic cardiomyopathy [15] has implicated cardiac fibrosis as a strong pathological contributor. Cardiac fibrosis leads to increased LV stiffness and decreased ventricular wall compliance, resulting in both systolic and in particular diastolic dysfunction [27]. The ECM remodeling plays an important role in cardiac fibrosis. The amount of extra cellular collagen is caused by an imbalance between synthesis and degradation. Our previous study [18], and others studies demonstrated that hyperglycemia increased MOS [28], stimulates cardiac fibroblasts expression TGF-β in vivo [29]. TGF-β is involved in the differentiation of fibroblasts to myofibroblasts [30], and causes excessive collagen production, accompanied by an increase in α-SMA, a surrogate maker of fibroblasts to myofibroblasts. Here we showed that an increase oxidative stress and collagen deposition in STZ-induced diabetic rats hearts, was not only concomitant with an increase TGF-β and α-SMA expression, but both were attenuated by ALA treatment. Together, these finding indicated that oxidative stress in diabetic rat hearts may contribute to fibroblasts to myofibroblasts differentiation, with increased collagen Type I and Type III expression resulting in cardiac remodeling.

Matrix metalloproteinases (MMPs) belong to a family of enzymes with proteolytic activity that plays an important role in EC matrix degradation. Indeed MMP-2, is involved in the pathogenesis of a wide viriety of cardiovascular disorders [31,32]. Moreover, MMP-2 has recently been shown to be a direct mediator of ventricular remodeling and systolic dysfunction [33]. Several studies in vivo demonstrated that cardiac fibrosis in diabetic cardiomyopathy is associated with a decrease in MMP-2 expression/activity [34-36]. The activity of MMPs is tightly controlled by tissue inhibitors of MMPs (TIMPs), and changes in the MMP/TIMP ratio, therefore, may lead to changes in EC matrix. Our results, demonstrated that ALA treatment ameliorated cardiac dysfunction, decreased LV Type I and III collagen deposition, reduced TIMP-2 and increased MMP-2 activity in STZ-induced diabetic cardiomyopathy. It is likely that oxidative stress in diabetic rat hearts may contribute to cardiac remodeling partly through decreasing cardiac gelatinolytic activity of active- and pro- MMP-2 levels (as shown in Figure 4D), especially as there was a marked increase in gelatinolytic activity of active MMP-2 levels (as shown in Figure 4E, active MMP-2/Pro-MMP-2).

With respect to the signaling pathway potentially involved in these responses, we found that hyperglycemia caused activation of JNK and p38 MAPK in heart tissue. The major members of MAPKs found in cardiac tissue include ERK1/2, JNK and p38 MAPK and these are most strongly activated by oxidative stress, hyperglycemia and proinflammatory cytokines [37]. Several studies have shown that hyperglycemia induced JNK and p38 MAPK activations can be suppressed by antioxidants [38,39]. Furthermore, pharmacological inhibition of p38 MAPK signaling can decrease the collagen content in DCM [40,41]. Our results are in agreement with these studies, demonstrating enhanced activation JNK and p38 MAPK in DCM model, and increased TGF-β expression and decreased pro- and active-MMP2, all resulting in cardiac remodeling. Additionally our results show that ALA treatment can significantly ameliorate cardiac remodeling by suppressing the activation of JNK and p38 MAPK, decreasing the TGF-β expression and increasing pro and active –MMP2, all without altering prevailing the glucose concentration. Therefore, not only can oxidative stress and stress signaling play an important role in development of cardiac remodeling in DCM, but these effects can be reversed.

## Conclusion

Collectively, under STZ-induced diabetic conditions, cardiac fibrosis is associated with increased synthesis and decreased degradation of ECM. This is accompanied by increased differentiation of cardiac fibroblasts to myofibroblasts and reduced MMP-2 activity, concomitant with increased mitochondrial oxidative damage and elevated expression and activation of JNK, p38 MAPK and TGF-β. All these changes can be reversed by ALA, suggesting that ALA possesses therapeutic potential in the treatment of DCM.

## Abbreviations

α-SMA, Smooth muscle actin; ALA, Alpha-Lipoic acid; DCM, Diabetic cardiomyopathy; +dp/dt, Maximum rate of rise of left ventricle pressure; -dp/dt, Maximum rate of fall of left ventricle pressure; ECM, Extracellular matrix; GSH/GSSG, Reduced/oxidized glutathione ratio; HR, Heart rate; JNK, c-Jun N-terminal kinase; LV, Left ventricular; LVEDP, Left ventricular end-diastolic pressure; LVSP, Left ventricular end-systolic pressure; MDA, Malondialdehyde; MMP-2, Matrix metalloproteinase-2; MMPs, Matrix metalloproteinases; MOS, Mitochondrial oxidative stress; SNK, Student newman keuls test; SOD, Superoxide dismutase; STZ, Streptozotocin; TBS, Tris-buffered saline; TGF-β, Transforming growth factor-β; TIMP-2, Tissue inhibitor of metalloproteinase-1.

## Competing interests

All authors declare that they have no competing interests.

## Authors’ contributions

All authors fulfill the criteria for authorship. CL and DY contributed to design and coordinate the study, interpret the findings and draft the manuscript. LL and HL contributed to draft the protocol and the statistical analysis. All authors read and approved the final manuscript.
